# Folate receptor 1 (FOLR1) targeted chimeric antigen receptor (CAR) T cells for the treatment of gastric cancer

**DOI:** 10.1371/journal.pone.0198347

**Published:** 2018-06-06

**Authors:** Minsung Kim, Suhkneung Pyo, Chung Hyo Kang, Chong Ock Lee, Heung Kyoung Lee, Sang Un Choi, Chi Hoon Park

**Affiliations:** 1 Bio & Drug Discovery Division, Korea Research Institute of Chemical Technology, Daejeon, Republic of Korea; 2 School of Pharmacy, Sungkyunkwan University, Suwon City, Kyunggi-do, Republic of Korea; 3 College of Pharmacy, Chungnam National University, Daejeon, Republic of Korea; 4 Department of Medicinal Chemistry and Pharmacology, Korea University of Science and Technology, Daejeon, Republic of Korea; Saint Louis University School of Medicine, UNITED STATES

## Abstract

Gastric cancer is a malignancy that has a high mortality rate. Although progress has been made in the treatment of gastric cancer, many patients experience cancer recurrence and metastasis. Folate receptor 1 (FOLR1) is overexpressed on the cell surface in over one-third of gastric cancer patients, but rarely is expressed in normal tissue. This makes FOLR1 a potential target for chimeric antigen receptor (CAR) T cell immunotherapy, although the function of FOLR1 has not been elucidated. CAR are engineered fusion receptor composed of an antigen recognition region and signaling domains. T cells expressing CAR have specific activation and cytotoxic effects against cancer cells containing the target antigen. In this study, we generated a CAR that targets FOLR1 composed of a single-chain variable fragment (scFv) of FOLR1 antibody and signaling domains consisting of CD28 and CD3ζ. Both FOLR1-CAR KHYG-1, a natural killer cell line, and FOLR1-CAR T cells recognized FOLR1-positive gastric cancer cells in a MHC-independent manner and induced secretion of various cytokines and caused cell death. Conclusively, this is the first study to demonstrate that CAR KHYG-1/T cells targeting FOLR1 are effective against FOLR1-positive gastric cancer cells.

## Introduction

Immunotherapy for cancer has made considerable progress due to improved efficacy in chemotherapy-refractory blood and solid tumors from patients. Clinical trials using immunotherapy have been successful in the treatment of malignant tumors by blocking immune cell inhibitory signals or by redirecting T cells to target cancer cells [1]. In adoptive T cell immunotherapy for cancer, T cells isolated from a patient are manipulated and expanded in vitro, and then reinfused into the patient [2]. One of the main types of adoptive T cell immunotherapy is the use of chimeric antigen receptor (CAR) T cells. T cells are reintroduced into a patient after conversion from the patient’s T cells to CAR T cells that express the engineered receptor specific for a cancer target through a retrovirus or lentivirus, leading to effective anticancer activity [3]. CAR consist of a combination of target recognition and T cell activation regions. The target recognition region is typically derived from a single-chain variable fragment (scFv) of an antibody and T cell activation regions are composed of one or more intracellular signaling domains that induce persistence and effector functions in T cells [4]. CAR T cells exhibit cytotoxic effects against target cells by recognizing specific antigens on the surface of target cells in a major histocompatibility complex (MHC) independent manner. CAR T cell immunotherapy has been developed for two decades, beginning with first-generation CARs that combined scFv of antibodies with FcR γ or CD3ζ chains. Second and third-generation CARs were developed to have one or more costimulatory domains, such as CD28, CD137 (4-1BB), ICOS, and OX40 [5]. In addition, several types of CARs targeting different antigens have been constructed and their effectiveness has been verified in clinical trials [6].

While this strategy is highly effective against blood cancers, clinical application for solid cancer has lacked efficacy. Additional factors for solid tumors require consideration, including disease status, tumor burden, CAR T cell infiltration, and the recruitment and activation of other immune responses, such as inflammation and immunosuppression [7]. Although the therapeutic efficacy of all types of CAR T cells has not been elucidated, an important issue is the choice of a target antigen. These targets include epidermal growth factor receptor (EGFR), carcinoembryonic antigen (CEA), human epidermal growth factor receptor 2 (HER2), and mesothelin (MSLN) all of which are currently being investigated in clinical trials [8].

Folate receptor 1 (FOLR1), also known as folate receptor alpha and folate binding protein, is a glycosylphosphatidylinositol-linked protein. Although the function of FOLR1 is unclear, FOLR1 has a high affinity for folate and is capable of internalizing folate [9]. FOLR1 is found to be overexpressed in various epithelial malignancies including ovarian, breast, renal, and lung cancers [10]. FOLR1 in normal tissues is expressed only on the apical surfaces of polarized epithelial cells and is not exposed to the bloodstream. These properties show that FOLR1 is an attractive candidate as a target for cancer therapy.

Gastric cancer is one of the most malignant cancers, with an especially high incidence in East Asia [11]. Although gastric cancer therapies have been continuously developed, many patients experience tumor recurrence and metastasis. Chemotherapy is the main treatment for gastric cancer, but it is less efficient and has systemic toxicity because of its nonspecific antitumor effects. FOLR1 can be a key immunotherapy target against gastric cancer because more than one-third of patients with gastric cancer have FOLR1 expression [12]. A clinical study using FOLR1-CAR T cells in patients with ovarian cancer showed that the FOLR1-CAR approach performed well with respect to safety [13]. These data suggest that FOLR1 can be a suitable target for CAR T cell immunotherapy in gastric cancer. In this study, we designed FOLR1-CAR composed of anti-FOLR1 scFv, CD28 and CD3ζ and evaluated the antitumor activity of CAR-mediated T cells against FOLR1 positive and negative gastric cancer cells.

## Materials and methods

### Ethics statement

Human peripheral blood mononuclear cells (PBMCs) were obtained from three healthy donors who provided written informed consent according to protocols approved by Korea National Institute for Bioethics Policy Institutional Review Board (approval no. P01-201607-31-003). NOD-SCID IL2R γ null (NSG) mice were obtained from Charles River Laboratories Japan, Inc. All of the procedures were approved by the Laboratory Animal Care and Use Committee of the Korea Research Institute of Chemical Technology and were conducted in accordance with the Institute for Laboratory Animal Research Guide for the Care and Use of Laboratory Animals. All efforts were made to minimize animal suffering.

### Cell culture

K562, Jurkat, MKN1, SNU216, SNU601, and SNU668 cells were obtained from the Korean Cell Line Bank. KHYG-1 cells were purchased from DSMZ (Germany). MKN7 and GCIY cells were purchased from RIKEN BioResource Center (Japan). RPMI-1640 and fetal bovine serum (FBS) were purchased from Hyclone (USA). All cells were maintained in RPMI-1640 supplemented with 10% FBS in a humidified incubator at 37°C with 5% CO_2_. PBMCs from healthy donors were expanded with Dynabeads Human T-Activator CD3/CD28 (Cat. #111.31D, Thermo Fisher Scientific, USA) for 2 weeks to obtain primary T cells. KHYG-1 cells and T cells were supplemented with 200 IU/ml and 500 IU/ml of human IL-2 (Cat. 202-IL, R&D systems, USA), respectively, in the culture medium.

### Cell transfection and infection

The FOLR1-CAR DNA, which contained the scFv of the FOLR1 antibody, CD28, and CD3ζ, was synthesized from Macrogen (Korea). Lentiviral expression vectors, including pLVX-Puro (Cat. #632164) and pLVX-IRES-ZsGreen1 (Cat. #632187), were obtained from Clontech (USA). Lentiviral packaging plasmids, including pMDLg/pRRE (Cat. #12251), pRSV-Rev (Cat. #12253), and pMD2.G (Cat. #12259), were purchased from Addgene (USA). The FOLR1-CAR DNA was inserted into the pLVX-puro and pLVX-IRES-ZsGreen vectors. Jurkat and human T cells were transfected with FOLR1-CAR vectors using the Neon Transfection System (Thermo Fisher Scientific, USA) according to the manufacturer’s instructions. The 293T cells were transfected with the following vectors: FOLR1-CAR, pMDLg/pRRE, pRSV-Rev, and pMD2.G for the production of lentivirus. After 48 h, the culture medium was harvested and centrifuged at 500 x g for 5 min. The centrifuged supernatant was passed through a 0.45-μm filter and concentrated using the Lenti-X Concentrator (Clontech, USA). KHYG-1 cells were incubated with lentivirus for 48 h and then the culture medium was replaced. Cells were selected for 2 weeks with puromycin (Sigma-Aldrich, USA) at a concentration of 2 μg/ml.

### Enzyme-linked immunosorbent assay (ELISA)

Jurkat cells and human T cells (2×10^5^) were co-cultured with target cells at an effector-target (E/T) ratio of 10:1 for 24 h and 4 h, respectively. Supernatants of the co-cultured cells were harvested and evaluated cytokine levels, including IL-2, IFN-γ, TNF-α, and GM-CSF, using ELISA kits (Biolegend, USA). Granzyme B was measured by ELISA kits (R&D systems, Cat. #DY2906-05). All cytokines were performed according to the operating manuals.

### Western blot assay

For western blotting, samples were harvested in RIPA buffer, separated via 10% SDS-PAGE, and then transferred to a nitrocellulose membrane. The membrane was incubated with the antibodies and detected by ECL reagent (Cat. #32209, Thermo Fisher Scientific, USA). The images were analyzed using the LAS-3000 Imager and Image Lab software. Densitometry of western blot bands was performed using ImageJ. Antibody information is provided in S1 Table.

### Imaging flow cytometry assay

Approximately 2×10^5^ cells were washed with PBS and resuspended in cold PBS supplemented with 10% FBS and 0.02% sodium azide. One μg of primary antibody was added and the cells were incubated for 30 min on ice. After washing with PBS, 1 μg of secondary antibody was added and the cells were incubated for 30 min on ice. Cells were washed with PBS and resuspended in 100 μl of Hoechst 33342 (Cat. #H3570, Thermo Fisher Scientific, USA) or DAPI (Cat. #D3571, Thermo Fisher Scientific, USA) counterstaining solution and incubated for 15 min at 37°C. Cells were analyzed using the NucleoCounter NC-3000 (ChemoMetec, Denmark) and data were acquired by NucleoView NC-3000 software. Antibody information is provided in S1 Table.

### Cell cytotoxic assay

KHYG-1 cells or human T cells were co-cultured with target cells (2×10^4^) transfected with the luciferase gene (Cat. #E1310, Promega, USA) at indicated E/T ratios for 4 h. Luciferase activity was detected by the Bright-Glo Luciferase Assay System (Promega, USA). The luciferase reagent was added to each well at a volume equal to the cell culture medium. The plates were shaken for 5 min to lyse the cells. Luminescence was measured on an EnVision reader (PerkinElmer, USA). To investigate the cytotoxicity pathway, 100 μM of Z-AAD-CMK (Cat. #BML-P165-0001, Enzo Life Sciences, USA) or 0.1 μg/ml of neutralization antibodies, anti-hFas ligand (Cat. #MAB126, R&D systems, USA) and anti-hTNF-α (Cat. #AF-210, R&D systems, USA), were added to the co-cultured cells.

### T cell proliferation assay

T cells (1×10^4^) were seeded in 96-well plates and incubated at 37°C with 5% CO_2_. An equal volume of CellTiter-Glo (Promega, USA) was added to each well and plates were incubated on a shaker at room temperature for 10 min. Luminescence was measured at intervals of 2 to 3 days using an EnVision reader (PerkinElmer).

### Xenograft studies

Six- to eight-week-old female NOD-SCID IL2R γ null (NSG) mice were obtained from Charles River Laboratories Japan, Inc. MKN1 cells (1×10^7^ cells/mouse) were subcutaneously injected into the right flank. Ten mice with tumor sizes reaching an average volume of approximately 100 mm^3^ were randomly divided into two groups (n = 5). On days 0 and 7, mice were treated with an intratumoral injection of mock or FOLR1-CAR KHYG-1 cells (1×10^7^ cells/mouse). Tumors were measured at intervals of 2 to 4 days using calipers, and the volume was calculated using the formula: volume = length × width^2^ × 0.5. The body weight of the mice was measured when the tumor volume was measured.

### Statistical analysis

All results are reported as the mean ± standard deviation (SD), and were analyzed using Prism 5.0 (GraphPad Software). Statistical comparison between two groups was analyzed using the two-sided unpaired Student’s t test. Statistical significance is indicated as follows: *P < 0.05, **P < 0.01, and ***P < 0.001.

## Results

### Identification and specific activity of a FOLR1-targeted CAR construct

**Fig 1:**
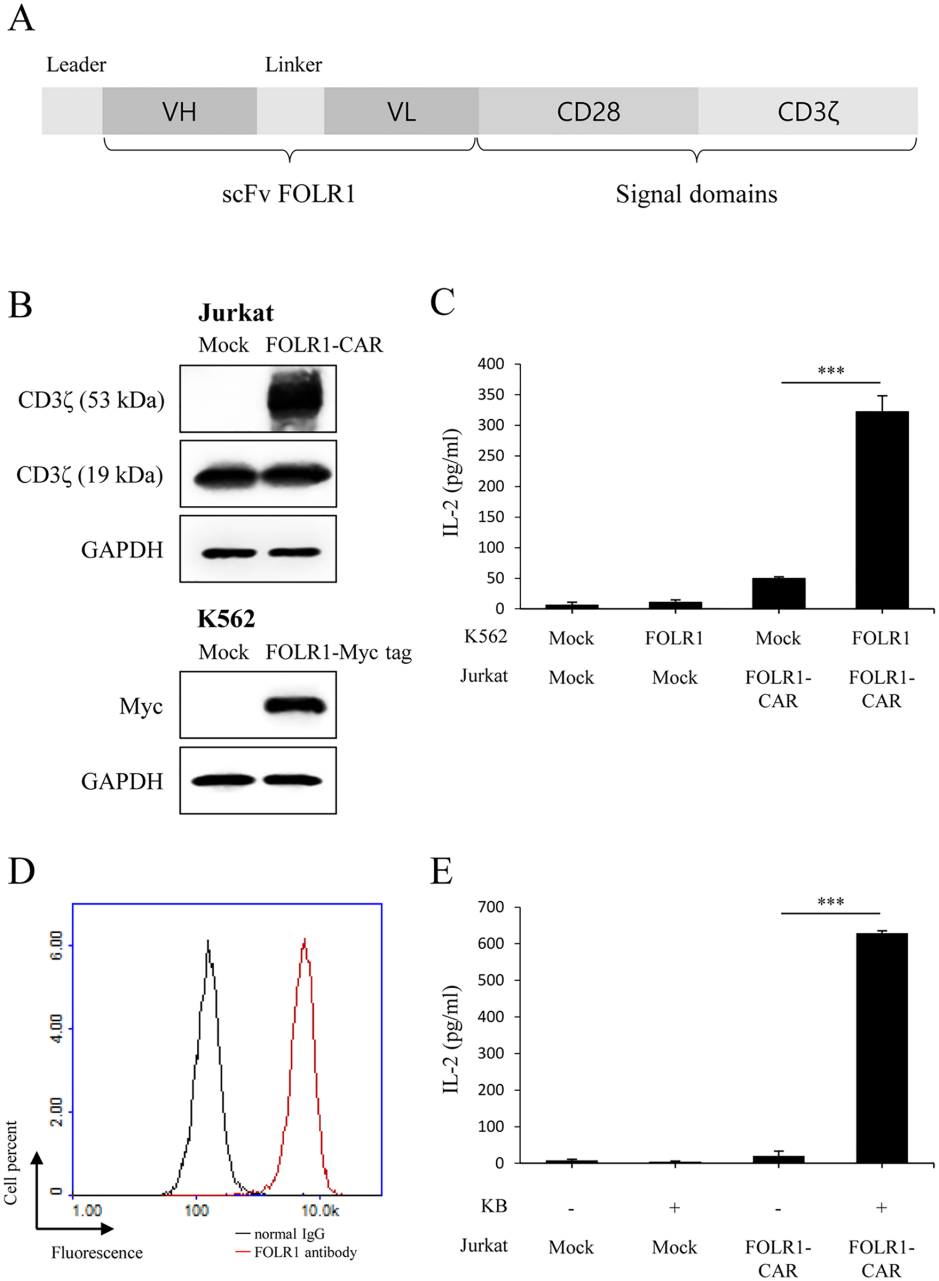
Schematic representation of the FOLR1-CAR construct and the specific activity against target cells expressing FOLR1. (A) A FOLR1-CAR gene consisting of a FOLR1-specific scFv, CD28 and CD3ζ was constructed. (B) The FOLR1-CAR vector was transfected into Jurkat cells, and the Myc tagged FOLR1 gene was transfected into K562 cells. FOLR1-CAR and FOLR1-Myc tag vectors were measured by western blot using CD3ζ and myc antibody, respectively. (C) FOLR1-CAR Jurkat cells were co-cultured with transfected K562 cells at an effector-target (E/T) ratio of 10:1 for 24 h. The levels of IL-2 were measured by enzyme-linked immunosorbent assay (ELISA). (D−E) The specific activity of Jurkat cells transfected with the FOLR-CAR vector was analyzed by co-culture with FOLR1-positive KB cells. (D) Expression of FOLR1 in KB cells was measured using FACS. (E) The levels of IL-2 secreted by FOLR1-CAR Jurkat cells were measured by ELISA after co-cultured with KB cells at an E/T ratio of 10:1 for 24 h. Experiments were repeated at least three times with similar results. The data are represented as the mean cytokine concentrations ± SD (pg/ml) from triplicate cultures. Statistical analysis was performed using the two-sided unpaired Student’s t test (***P<0.001).

We constructed a second-generation CAR vector expressing a fusion protein composed of a single-chain variable fragment (scFv) of FOLR1 antibody and signaling domains consisting of CD28 and CD3ζ (Fig 1A). Jurkat cells, a CD4-positive T cell line, are commonly used to study T cell receptor (TCR) signaling because they secrete IL-2 when activated. To verify the specific activity of FOLR1-CAR against FOLR1 expressing cells, we transfected the CAR construct into Jurkat cells and transfected the FOLR1 gene into the FOLR1-negative cell line, K562 (Fig 1B). In western blot analysis, endogenous CD3ζ was detected at 19 kDa, but CD3ζ was detected at about 53 kDa in our FOLR1-CAR construct-positive cells. Jurkat cells that were mock-transfected or transfected with the FOLR1-CAR gene were co-cultured with K562 cells that were either mock-transfected or transfected with the FOLR1 gene at an effector-target (E/T) ratio of 10:1 for 24 h. Secretion of the cytokine IL-2 was measured in order to determine whether or not there was activation. Levels of IL-2 were significantly increased when FOLR1-CAR Jurkat cells were co-cultured with FOLR1-transfected K562 cells compared to when co-cultured with mock-transfected K562 cells (Fig 1C). To determine whether the FOLR1-CAR Jurkat cells specifically recognize the FOLR1-positive cells, we used KB cells which are one of the subline of HeLa cells (keratin forming tumor cell line HeLa) and are commonly used in FOLR1 studies. When FOLR1 expression was measured using fluorescence-activated cell sorting (FACS), KB cells strongly expressed FOLR1 (Fig 1D). The transfected Jurkat cells were co-cultured with KB cells at an E/T ratio of 10:1 for 24 h. FOLR1-CAR Jurkat cells secreted significantly higher levels of IL-2 when co-cultured with KB cells (Fig 1E). These results indicate that our FOLR1-CAR can recognize FOLR1 on the cell surface and functions well against cells expressing FOLR1.

### FOLR1-CAR Jurkat cells were specifically activated by FOLR1-positive gastric cancer cells

**Fig 2:**
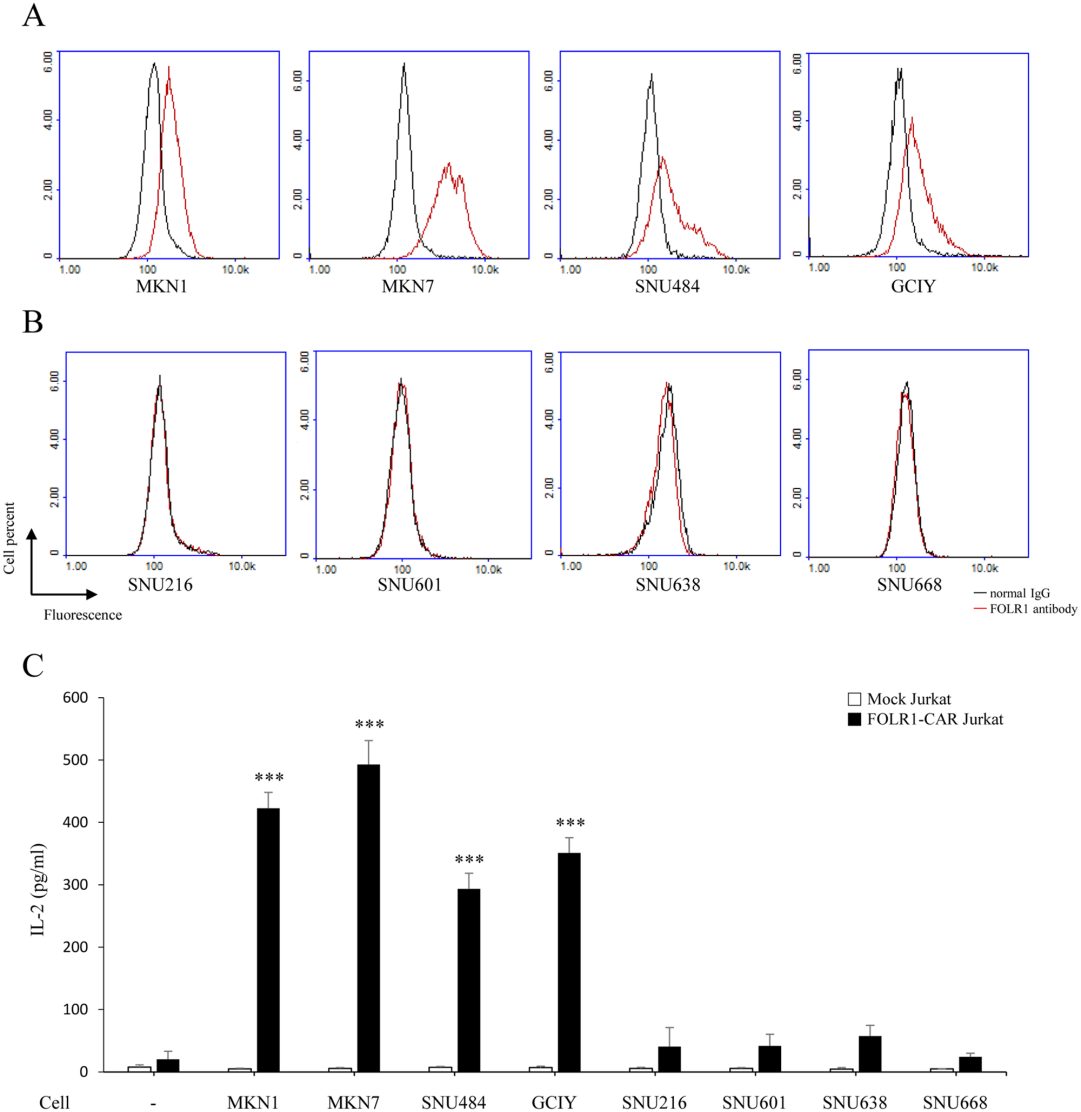
Specific activity of FOLR1-CAR Jurkat cells against FOLR1-positive GC cells. FACS was used to measure the surface expression of FOLR1 in a series of human GC cell lines, including MKN1, MKN7, SNU216, SNU484, SNU601, SNU638, SNU668, and GCIY. GC cell lines were separated into (A) FOLR1-positive GC cell lines (MKN1, MKN45, SNU484, and GCIY) and (B) FOLR1-negative GC cell lines (SNU216, SNU601, SNU638 and SNU668) based on the FACS results. (C) GC cell lines were co-cultured with FOLR1-CAR Jurkat cells at E/T ration of 10:1 for 24 h. After incubation, the levels of IL-2 were measured by ELISA. Experiments were repeated at least three times with similar results. The data are represented as the mean cytokine concentrations ± SD (pg/ml) from triplicate cultures. Statistical analysis was performed using the two-sided unpaired t-test and data were compared to FOLR1-CAR Jurkat cells cultured alone (***P<0.001).

To evaluate the effect of FOLR1-CAR against gastric cancer (GC) cells, we divided them into FOLR1-positive and FOLR1-negative cells. FACS was used to assess surface expression of FOLR1 in a series of human GC cells. FOLR1-positive cells were MKN1, MKN7, SNU484, and GCIY (Fig 2A), while FOLR1-negative cells were SNU216, SNU601, SNU638, and SNU668 (Fig 2B). To determine the specific activity of FOLR1-CAR Jurkat cells against FOLR1-positive or FOLR1-negative GC cells, transfected Jurkat cells were co-cultured with GC cells at an E/T ratio of 10:1 for 24 h. The levels of IL-2 were significantly elevated when FOLR1-CAR Jurkat cells were co-cultured with FOLR1-positive cells (MKN1, MKN7, SNU484, and GCIY) (Fig 2C). However, when FOLR1-CAR Jurkat cells were co-cultured with FOLR1-negative GC cells (SNU216, SNU601, SNU638, and SNU668), there was no difference in the levels of IL-2 when comparing to FOLR1-CAR Jurkat cells cultured alone. Taken together, our results suggest that the FOLR1-CAR construct specifically recognized FOLR1-positive GC cells and induced Jurkat cells activation.

### Expression of FOLR1-CAR in KHYG-1, a natural killer cell line, has strong cytotoxic effects against FOLR1-positive GC cells

**Fig 3:**
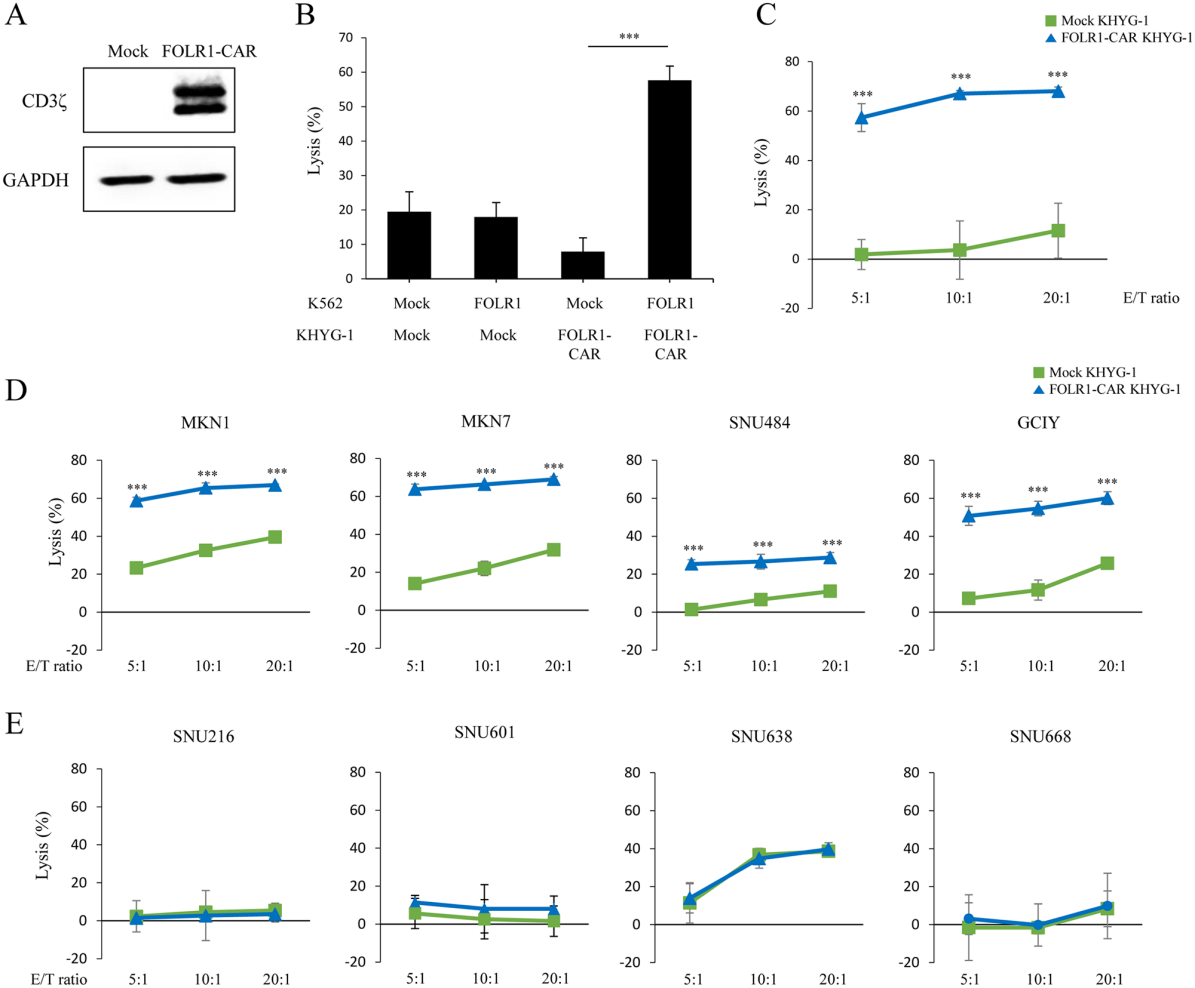
Specific cytotoxicity of FOLR1-CAR KHYG-1 cells against FOLR1-positive cells. KHYG-1 cells were infected with lentivirus encoding the FOLR1-CAR or mock vector and were selected with puromycin for 2 weeks. (A) FOLR1-CAR expression was measured by western blot using the CD3ζ antibody in KHYG-1 cells. (B) The cytotoxic effects of KHYG-1 cells on K562 cells transfected with FOLR1 or mock vector were measured by luciferase assay at an E/T ratio of 10:1 for 4 h. (C) The lysis percentages of mock KHYG-1 and FOLR1-CAR KHYG-1 cells against KB cells were determined in a dose-dependent manner for 4 h using the luciferase assay. (D−E) To determine the lysis percentages of FOLR1-CAR KHYG-1 cells against GC cells, mock KHYG-1 and FOLR1-CAR KHYG-1 cells were co-cultured with (D) FOLR1-positive GC cells or (E) FOLR1-negative GC cells at an indicated E/T ratio for 4 h. Experiments were repeated at least three times with similar results. The data are represented as the mean rate of lysis ± SD (%) from triplicate cultures. Statistical analysis was performed using the two-sided unpaired t-test (***P<0.001).

To identify FOLR1-CAR function in cytotoxic cells, we used KHYG-1 cells, a natural killer (NK) cell line with strong cytotoxic activity against target cells. KHYG-1 cells were infected with lentivirus expressing FOLR1-CAR or a mock vector and then selected with puromycin for 2 weeks. Stable FOLR1-CAR expression in KHYG-1 cells was measured by western blot analysis (Fig 3A). The luciferase assay was performed using target cells transfected with the luciferase gene to evaluate the percentage of lytic target cells. K562 cells were either mock or FOLR1 gene co-transfected with the luciferase gene and they were then co-cultured with KHYG-1 cells at an E/T ratio of 10:1 for 4 h. FOLR1-CAR KHYG-1 cells had high cytotoxicity against FOLR1 K562 cells compared to mock-transfected K562 cells (Fig 3B). In addition, when KHYG-1 cells were co-cultured with KB cells at the indicated E/T ratios for 4 h, FOLR1-CAR KHYG-1 showed higher average killing activity against KB cells compared to mock KHYG-1 cells (Fig 3C). The cytotoxicity of FOLR1-CAR KHYG-1 cells was increased as the E/T ratio increased and KB cell death was clearly observed at E/T ratio 10:1 (S1 Fig). To investigate the cytotoxic effects of FOLR1-CAR KHYG-1 cells against GC cells, KHYG-1 cells were co-cultured with GC cells for 4 h. There was an increase in FOLR1-positive GC cells lysis in the presence of FOLR1-CAR KHYG-1 cells when comparing to cells in the presence of the mock KHYG-1 cells (Fig 3D). However, FOLR1-CAR KHYG-1 and mock KHYG-1 cells showed similar lytic activity against FOLR1-negative GC cells (Fig 3E). Hence, we demonstrated that FOLR1-CAR KHYG-1 cells have potent cytotoxic effects against FOLR1-positive GC cells.

### Expression of the FOLR1-CAR gene in T cells using lentiviral infection

**Fig 4:**
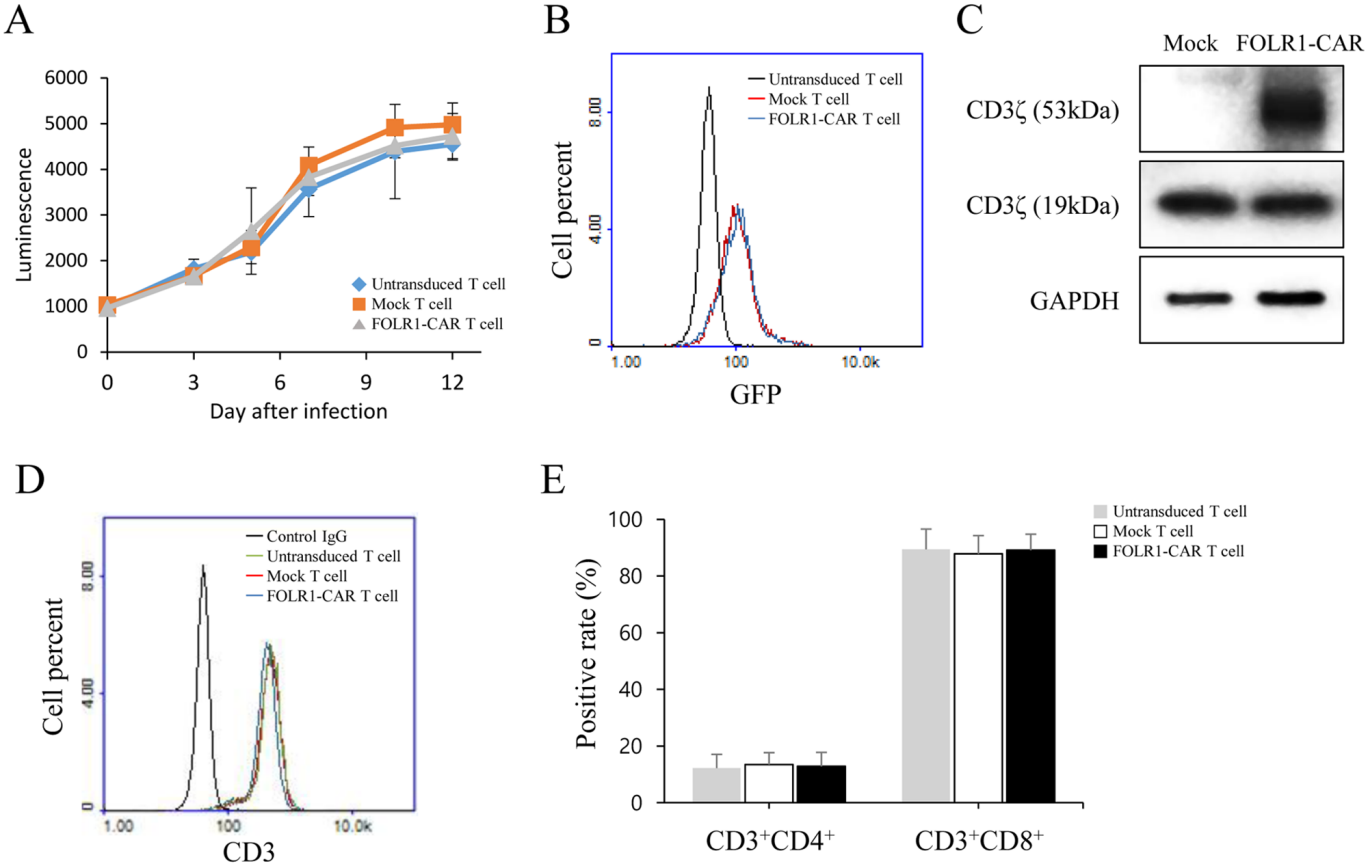
Generation and characterization of FOLR1-CAR T cells. T cells were obtained from human PBMCs of three healthy donors and the FOLR1-CAR gene was cloned into the pLVX-IRES-GFP vector and the lentivirus infection efficiency was determined. (A) The growth rate of infected or untransduced T cells was measured by CellTiter-Glo assay for 12 days. (B) Lentivirus infection rate was analyzed by GFP fluorescence on day 12 using FACS analysis. (C) Expression of FOLR1-CAR in T cells was measured by western blot analysis. (D-E) The population of T cells was evaluated by FACS analysis using CD3, CD4, and CD8 antibodies on day 12. Experiments were repeated three times with similar results. The data are represented as the mean of luminescence ± SD and positive rate ± SD (%) from triplicate cultures. Statistical analysis was performed using the two-sided unpaired t-test.

The results described above indicated specific activity of FOLR1-CAR NK cells against FOLR1-positive GC cells. To investigate the function of FOLR1-CAR T cells against FOLR1-positive cells, T cells were isolated from human peripheral blood mononuclear cells (PBMCs) of three healthy donors. T cells were infected with lentivirus containing FOLR1-CAR-IRES-GFP vector to evaluate the lentivirus infection efficiency. After lentivirus infection, the growth rate of T cells was measured by the CellTiter-Glo assay. The growth rate of infected T cells was not significantly different from that of untransduced T cells for 12 days (Fig 4A). Lentivirus infection rates were measured by GFP fluorescence in T cells using FACS analysis on day 12. Rates of infection for FOLR1-CAR- and mock-transfection were 70% and 69%, respectively, in infected T cells compared to those in untransduced T cells (Fig 4B). Additionally, FOLR1-CAR expression in T cells was measured by western blot analysis (Fig 4C). Using FACS analysis on day 12, we determined that T cells had nearly 100% CD3^+^ expression (Fig 4D). The T cells consisted mainly of a large population of CD8^+^ T cells and a small population of CD4^+^ T cells. There was no difference between the untransduced and uninfected groups in the T cell populations (Fig 4E).

### FOLR1-CAR T cells act specifically towards KB cells and granzyme B is a major factor in target cell death

**Fig 5:**
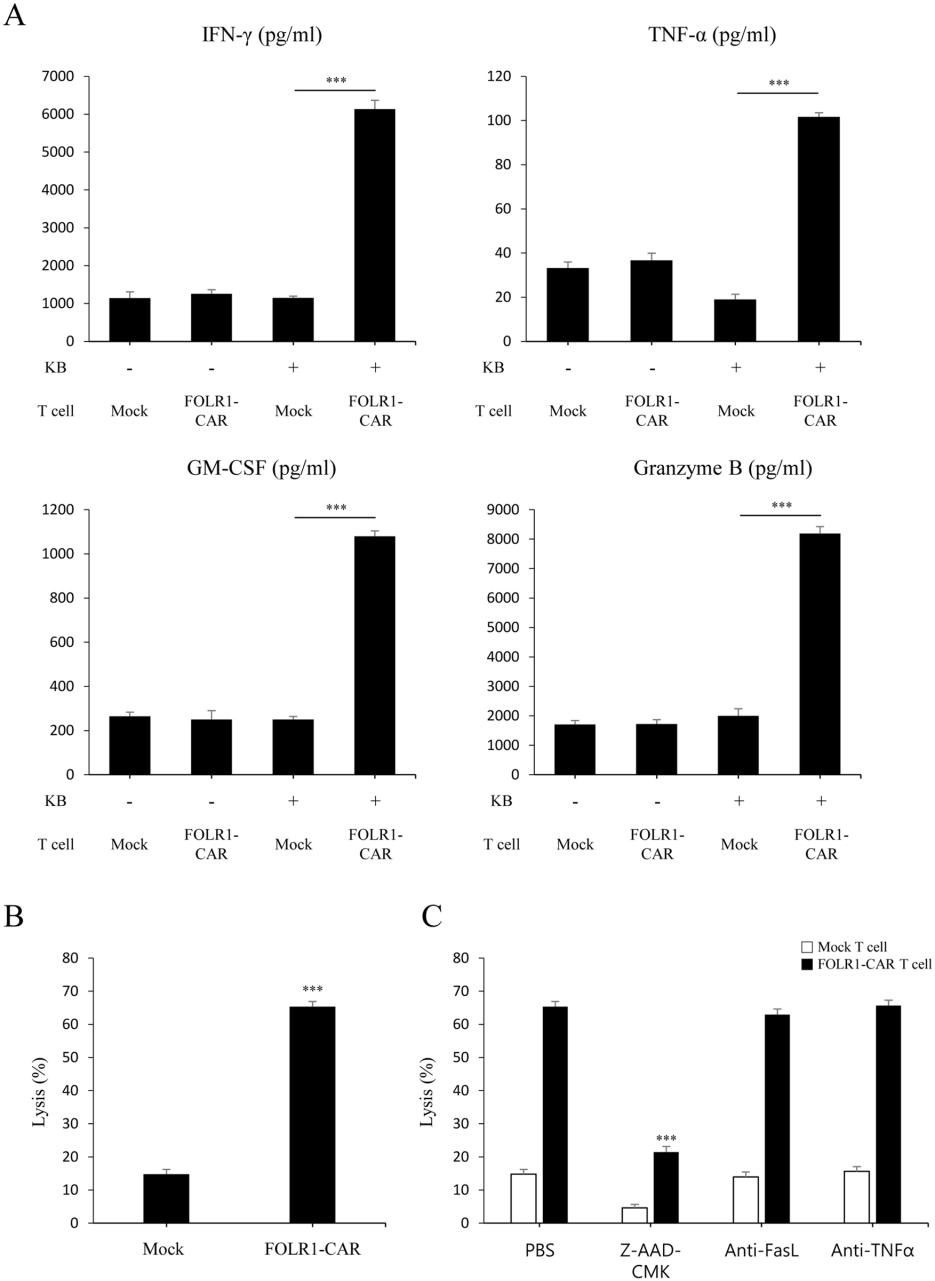
Specific activity of FOLR1-CAR T cells against KB cells and analysis of T cell-mediated cytotoxic factors. Mock or FOLR1-CAR T cells were co-cultured with KB cells at an E/T ratio of 10:1 for 4 h. (A) The levels of cytokines IFN-γ, TNF-α, GM-CSF, and granzyme B were measured by ELISA and (B) the cytotoxic effects were evaluated by luciferase assay. (C) To determine the factors that induced cell death, FOLR1-CAR T cells were co-cultured with KB cells in the presence of 100 μM of Z-AAD-CMK, 0.1 μg/ml of anti-FasL antibody, and anti-TNF-a antibody. Experiments were repeated at least three times with similar results. The data are represented as the mean cytokine concentrations ± SD (pg/ml) and rate of lysis ± SD (%) from triplicate cultures. Statistical analysis was performed using the two-sided unpaired t-test (***P<0.001).

To determine whether FOLR1-CAR T cells recognize cells expressing FOLR1, T cells were co-cultured with KB cells at an E/T ratio of 10:1 for 4 h. Compared with mock T cells, FOLR1-CAR T cells secreted significantly higher levels of the proinflammatory cytokines IFN-γ, TNF-α, GM-CSF, and granzyme B against KB cells (Fig 5A). Furthermore, FOLR1-CAR T cells showed higher average cytotoxicity against KB cells compared to mock T cells (Fig 5B) and KB cell death was clearly observed (S2 Fig). T cell cytotoxicity is mediated by two predominant pathways: (1) secretion of perforin and granzyme B and (2) activation of death receptor signaling via Fas/Fas ligand (FasL) or TNF-α/TNF-R [14]. To determine which pathways contributed to KB cell death by FOLR1-CAR T cells, KB cells were co-cultured with mock or FOLR1-CAR T cells in the presence of Z-AAD-CMK, a granzyme B inhibitor, an anti-FasL antibody for blocking the FasL pathway, or an anti-TNF-α antibody for neutralization. After 4 h incubation, Z-AAD-CMK significantly reduced lysis of KB cells in FOLR1-CAR T cells from 65.3% to 21.4% and in mock T cells from 14.7% to 4.5%, while anti-FasL antibody and anti-TNF-α had no effect (Fig 5C). These data suggest that KB cells are lysed by granzyme B dependent manner.

### FOLR1-CAR T cells are activated by the ZAP70 signaling pathway, similar to the TCR reaction, and induce target cell apoptosis

**Fig 6:**
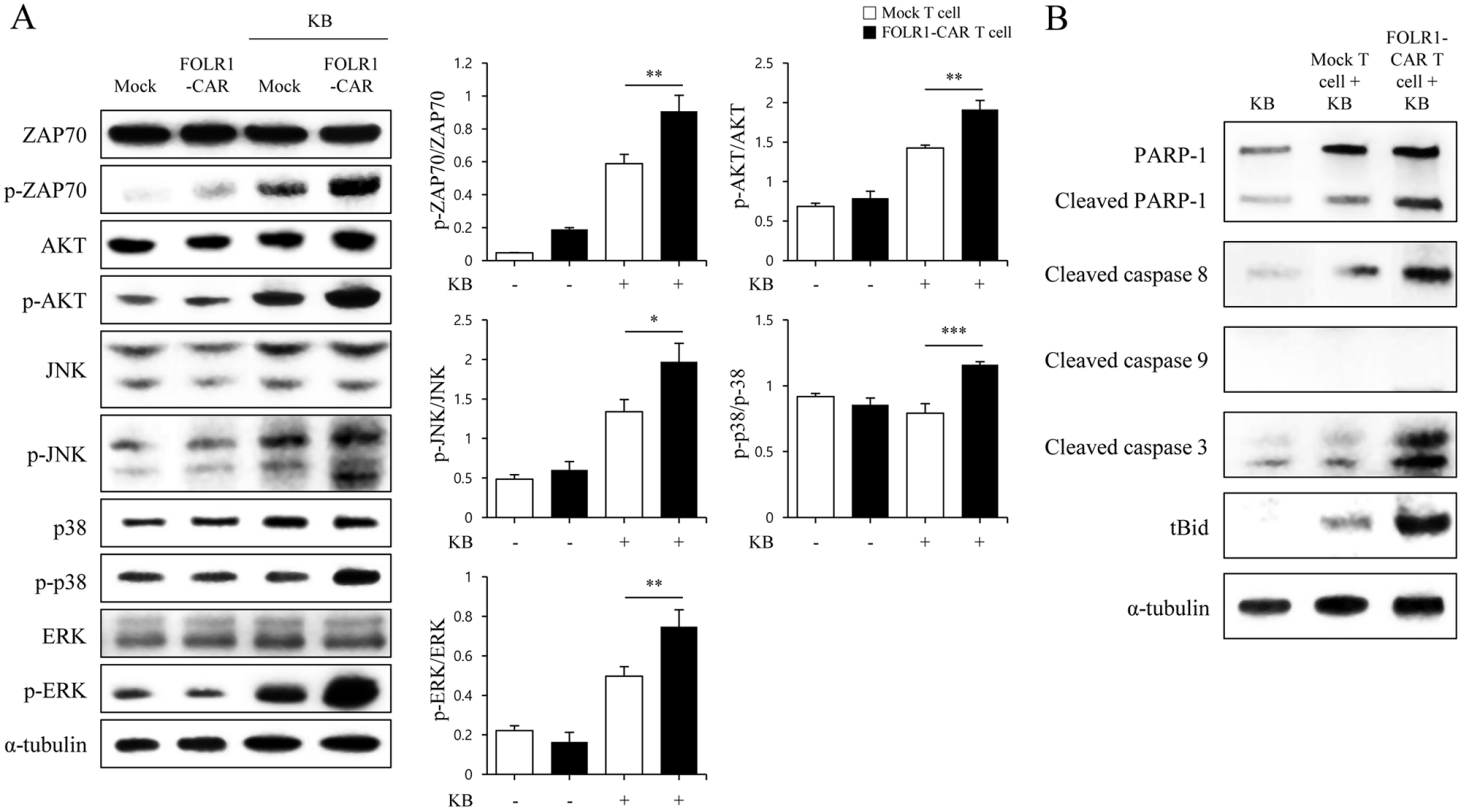
Specific activity of FOLR1-CAR T cells via ZAP70 signaling pathway is similar to the TCR reaction and KB cell death is caused by granzyme B. To investigate FOLR1-CAR T cell activation and KB cell death signaling, mock or FOLR1-CAR T cells were co-cultured with KB cells at an E/T ratio of 10:1 for 4 h. (A) ZAP70, AKT, JNK, p38, and ERK associated with TCR signaling were measured by western blot analysis in mock or FOLR1-CAR T cells when incubated alone or with KB cells. The graphs presented ratio of phosphorylation by quantification of the western blot bands from three independent experiments. The data are represented as the mean ratio of densitometry values ± SD. Statistical analysis was performed using the two-sided unpaired t-test (*P<0.05, *P<0.01, and ***P<0.001). (B) Pro-apoptotic proteins PARP, cleaved caspase 8, caspase 9, caspase 3, and tBid were measured in KB cells by western blot analysis. Experiments were repeated three times with similar results.

To identify the mechanism underlying FOLR1-CAR T cell activation and KB cell death, T cells were co-cultured with KB cells at an E/T ratio of 10:1 for 4 h. Most TCR reactions depend on the recruitment of endogenous downstream signaling proteins, such as zeta-chain-associated protein kinase 70 (ZAP70) [15]. As shown in Fig 6A, phosphorylation of ZAP70 was strongly induced in FOLR1-CAR T cells compared to mock T cells. This implies that FOLR1-CAR activation, like in the TCR reaction, is dependent on the catalytic activity of ZAP70. Phosphorylation of major factors for T cell activation associated with TCR signaling, including p-AKT, p-JNK, p-p38, and p-ERK, was measured by western blot analysis and was quantified by normalizing to the total proteins. Phosphorylation of AKT, JNK, p38, and ERK was significantly induced by KB cells in FOLR1-CAR T cells compared to in mock T cells. In addition, to investigate the mechanism of KB cell death by granzyme B of FOLR1-CAR T cells, we performed western blot analysis for apoptosis-related proteins in KB cells. Granzyme B, the secretory granules of cytotoxic T cells and NK cells, is a well-known serine protease. Once granzyme B is secreted into the target cells, it activates several pro-apoptotic proteins [16]. When KB cells were co-cultured with FOLR1-CAR T cells, cleaved PARP-1, caspase 3, caspase 8, and tBid were significantly increased in KB cells, but cleaved caspase 9, which is associated with the intrinsic apoptosis pathway, was not detected (Fig 6B). These data reveal that FOLR1-CAR activates T cell in a mechanism very similar to TCR reaction, and granzyme B, which is secreted by activated CAR T cells, induces apoptosis through the extrinsic pathway in target cells.

### FOLR1-CAR T cells specifically respond to FOLR1-positive GC cells

**Fig 7:**
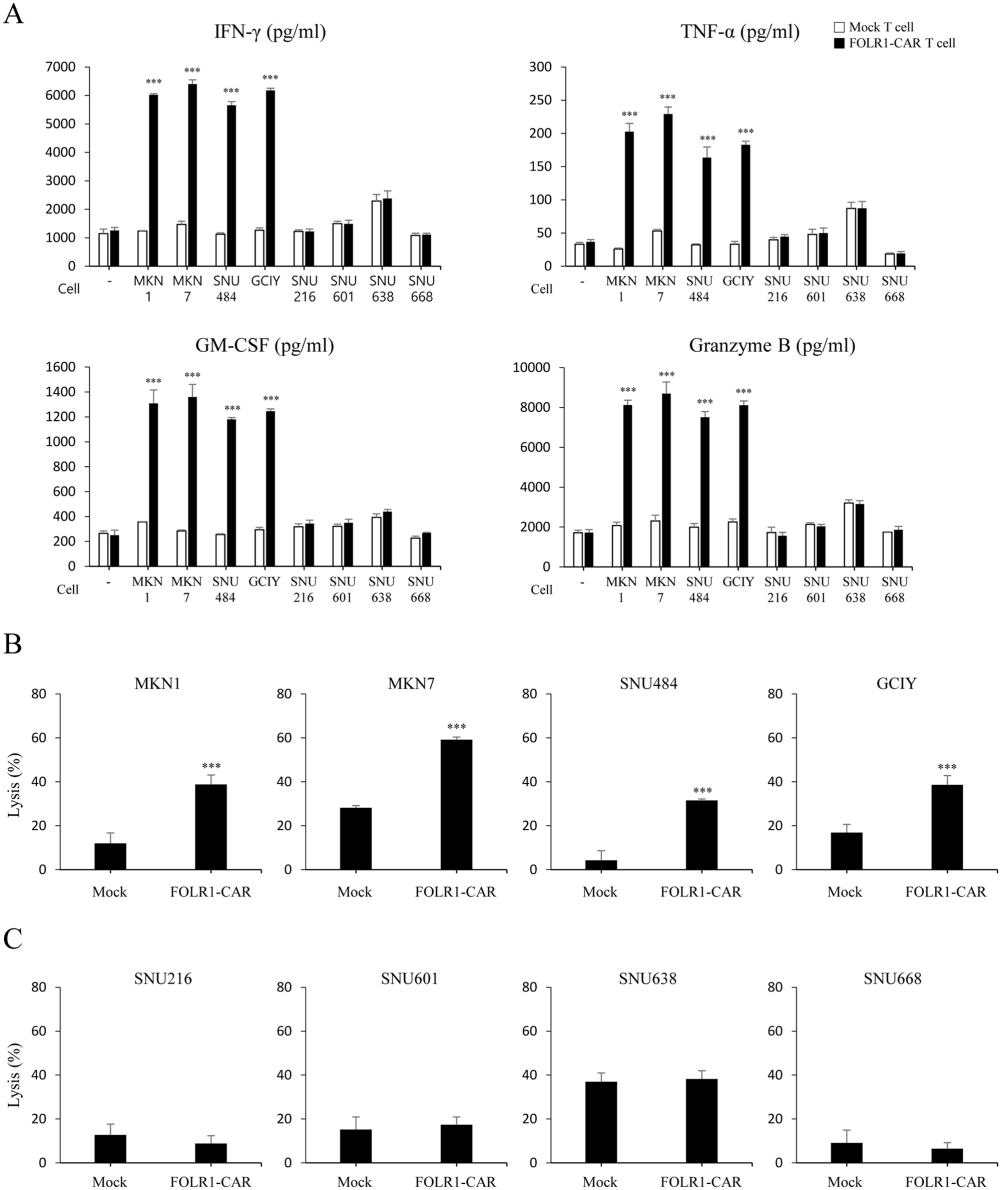
Specific response of FOLR1-CAR T cells against FOLR1-positive GC cells. T cells were co-cultured with FOLR1-positive GC cells (MKN1, MKN7, SNU484, and GCIY) or FOLR1-negative GC cells (SNU216, SNU601, SNU638, and SNU668) at an E/T ratio of 10:1 for 4 h. (A) The cytokine levels, including IFN-r, TNF-a, GM-CSF, and granzyme B were evaluated by ELISA. (B-C) The specific cytotoxicity of FOLR1-CAR T cells against FOLR1-positive GC cells (B) or FOLR1-negative GC cells (C) were measured by luciferase assay. Experiments were repeated three times with similar results. The data are represented as the mean cytokine concentrations ± SD (pg/ml) and rate of lysis ± SD (%) from triplicate cultures. Statistical analysis was performed using the two-sided unpaired t-test and data were compared with mock T cells. (***P<0.001).

**Fig 8:**
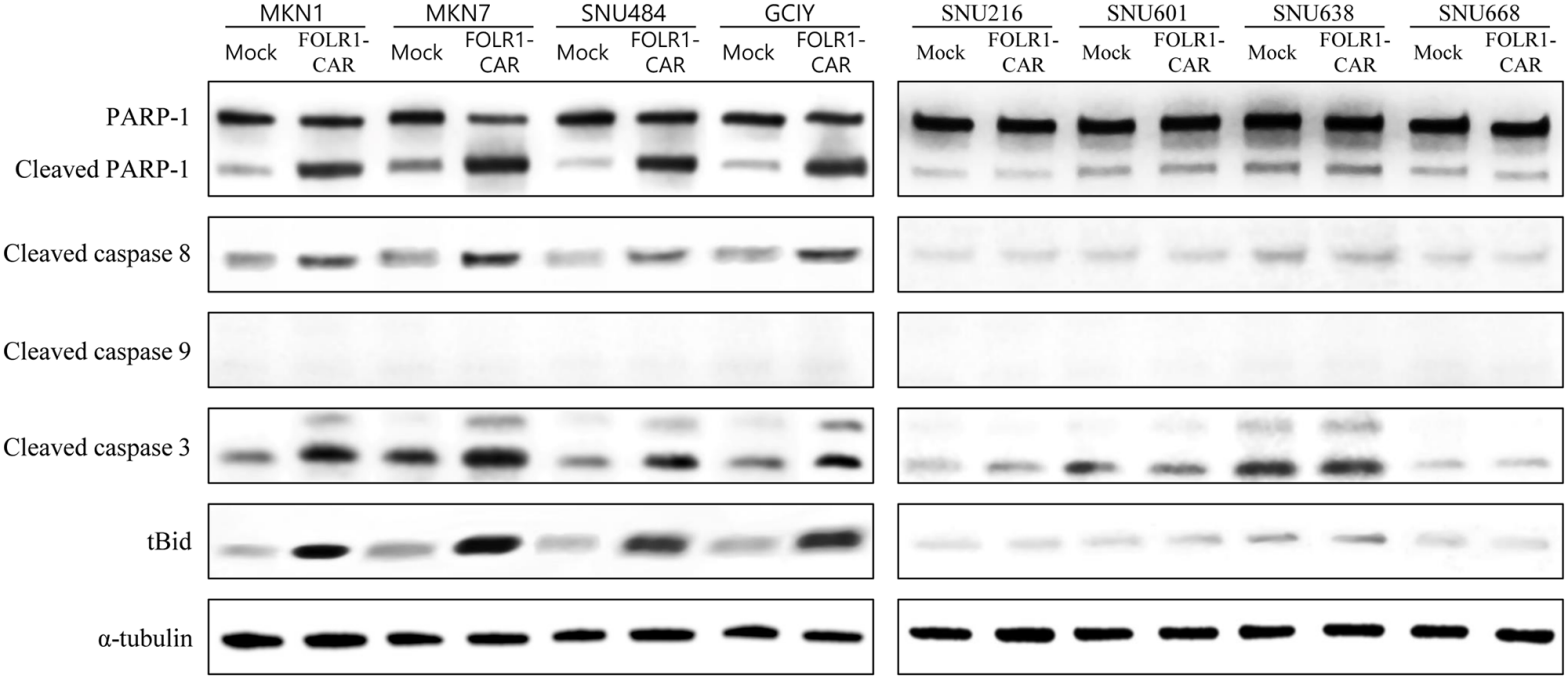
Analysis of pro-apoptotic proteins in GC cells co-cultured with T cells. T cells were co-cultured with FOLR1-positive GC cells (MKN1, MKN7, SNU484, and GCIY) or FOLR1-negative GC cells (SNU216, SNU601, SNU638, and SNU668) at an E/T ratio of 10:1 for 4 h. Pro-apoptotic proteins PARP, cleaved caspase 8, caspase 9, caspase 3, and tBid in FOLR1-positive or -negative GC cells were measured by western blot analysis. Experiments were repeated three times with similar results.

FOLR1-CAR T cells were co-cultured with GC cells and the specific activity of FOLR1-CAR T cells against FOLR1-positive or FOLR1-negative GC cells was measured at an E/T ratio of 10:1 for 4 h. The FOLR1-CAR T cells produced high levels of IFN-γ, TNF-α, GM-CSF, and granzyme B when they were co-cultured with FOLR1-positive GC cells (MKN1, MKN7, SNU484, and GCIY), whereas no significant elevation of cytokine levels was observed when the FOLR1-CAR T cells were co-cultured with FOLR1-negative GC cells (SNU216, SNU601, SNU638, and SNU668) compared to mock T cells (Fig 7A). In addition, we examined cytotoxicity of FOLR1-CAR T cells against GC cells using a luciferase assay. The cytotoxic effects of FOLR1-CAR T cells against FOLR1-positive GC cells were significantly higher than the mock T cells (Fig 7B). However, FOLR1-CAR T cells did not induce cell death compared to mock T cells in FOLR1-negative GC cells (Fig 7C). Furthermore, western blot analysis was performed on apoptosis-related proteins in GC cells. Similar to Fig 6B, pro-apoptotic proteins were clearly increased when co-cultured with FOLR1-CAR T cells in FOLR1-positive GC cells, but FOLR1-negative GC cells showed no difference when co-cultured with mock or FOLR1-CAR T cells (Fig 8). Additionally, cleaved caspase 9 was not detected in either condition. These data indicate that FOLR1-CAR T cells specifically react with FOLR1-positive GC cells and induce apoptosis in GC cells.

### FOLR1-CAR KHYG-1 cells have antitumor activity against FOLR1-positive MKN1 cells *in vivo*

**Fig 9:**
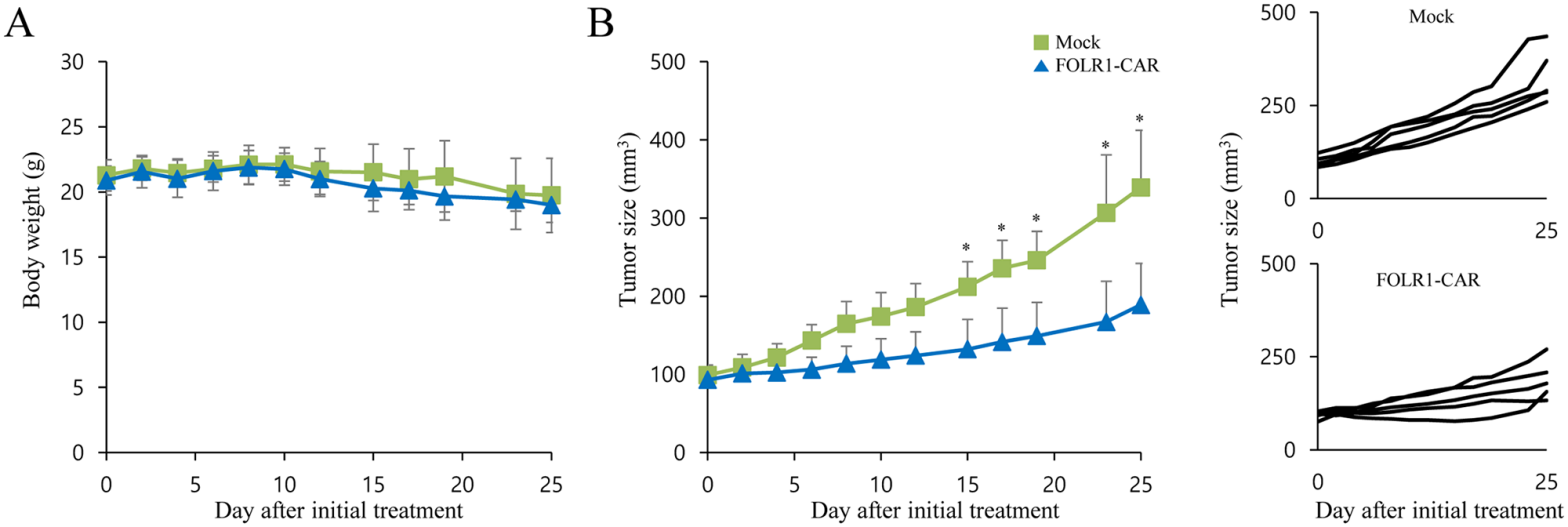
Antitumor efficacy of FOLR1-CAR KHYG cells against xenografts derived from FOLR1-positive MKN1 cells in mice. Ten NOD-SCID IL2R γ null (NSG) mice were subcutaneously implanted with MKN1 cells for approximately 14 days until tumor volume reached approximately 100 mm^3^ and then randomly divided into two groups (n = 5). The mice were injected intratumorally with either mock or FOLR1-CAR KHYG-1 cells on days 0 and 7. Body weight (A) and tumor size (B) were measured simultaneously at intervals of 2 to 4 days. Line graphs represented an individual animal. Experiments were repeated two times with similar results. The data are represented as the mean body weight ± SD (g) and the mean tumor size ± SD (mm^3^). Statistical analysis was performed using the two-sided unpaired t-test (*P<0.05).

One of the FOLR1-positive GC cell lines, MKN1 cells, is a well-established xenograft model of GC for therapeutic studies. To determine the antitumor ability of FOLR1-CAR NK cells *in vivo*, MKN1 cells were subcutaneously injected into in NOD-SCID IL2R γ null (NSG) mice. When the tumor size reached approximately 100 mm^3^ after inoculation, the mice were divided into two groups and treated with mock or FOLR1-CAR KHYG-1 cells twice on day 0 and 7. The body weight and tumor size of the mice were measured at intervals of 2 to 4 days for 25 days after initial treatment. During the treatment period, there was no significant difference in weight change between mice treated with mock KHYG-1 (19.74 ± 2.85 g) and FOLR1-CAR KHYG-1 cells (19.01 ± 1.32 g) groups on day 25 (Fig 9A). Additionally, there was no change in the weight of mice treated with mock and FOLR1-CAR KHYG-1 cells on day 25 compared to day 0 (20.88 ± 1.12 g). Compared with mock KHYG-1 cells, tumor growth was significantly reduced by FOLR1-CAR KHYG-1 cells at day 15 (Fig 9B). On day 25, the sizes of tumors treated with mock or FOLR1-CAR KHYG-1 cells groups were 338.94 ± 73.16 mm^3^ and 188.85 ± 53.10 mm^3^, respectively.

## Discussion

Although there have been many studies on CAR T cells immunotherapy, there have been few studies using CAR T cells to target GC cells. To our knowledge, this is the first study showing that FOLR1-CAR cells have specific and effective cytotoxic effects against FOLR1-positive GC cells. GC is associated with poor clinical outcomes and a high mortality rate, which has been difficult to improve. Several markers of GC cells have been discovered and examined in preclinical studies. Current clinical trials involving CAR T cell therapy in GC patients are targeting HER2, CEA, Mucin-1, and epithelial cell adhesion molecule (EpCAM) [17]. They are widely and highly expressed in various cancers, including GC, and are rarely expressed on the surface of normal tissues. Such properties make these proteins ideal targets for CAR T cell therapy. Several preclinical studies were performed with CAR T cells targeting HER2, CEA, Mucin-1, and EpCAM, and results showed significant antitumor activity against GC cells expressing the target [18–21].

FOLR1 has been used extensively as a key target in immunotherapy against various solid tumors since it is reported to be overexpressed in tumors. On the other hand, the distribution of FOLR1 in normal human tissues is restricted to low-level expression. FOLR1 has a high affinity for folic acid and is thought to initiate intracellular regulatory signaling upon binding with folate. FOLR1 that is bound to folic acid was shown to be internalized, entered the nucleus, and acted as a transcription factor [22]. FOLR1 knockdowns in ovarian cancer cells inhibited folate-mediated cellular proliferation and suppressed an invasive phenotype [23].

A variety of FOLR1 targeting approaches, including folate-drug conjugates, monoclonal antibodies, and T cell therapies, have been developed for the treatment of cancer [24]. Vintafolide, a conjugate of folate and the drug desacetylvinblastine hydrazide, uses the self-immolative disulfide-linker system and effectively penetrates into the cytoplasm of cancer cells through FOLR1 [25]. Farletuzumab, a monoclonal antibody targeting FOLR1, induces the death of cancer cells expressing FOLR1 by antibody-dependent cellular cytotoxicity (ADCC), complement-dependent cytotoxicity (CDC), and inhibition of Lyn kinase substrate phosphorylation [26–28]. In addition, the first clinical study using FOLR1-CAR T cells in patients with ovarian cancer has shown that it has performed well in terms of safety and efficacy [13]. Therefore, FOLR1 is an attractive candidate for the targeted treatment of cancer and the FOLR1 targeting strategies can increase treatment success rates.

We constructed a CAR containing CD28 and CD3ζ signaling domains (Fig 1A). In most CAR studies, FcR γ or CD3ζ chain was used as the stimulatory domain and they have the immunoreceptor tyrosine-based activation motifs [29, 30]. Several studies demonstrated that CAR T cells equipped with CD3ζ, compared to FcR γ, had effective cytotoxicity against tumor cells [31, 32]. In addition, second-generation CAR has the additional co-stimulatory domains necessary to enhance CAR T cell activation and proliferation. Despite the presence of several alternative co-stimulatory domains, CD28 was the most effective in T cells and most of the modular designs used in clinical trials are based on the CD28 signaling domain [6, 33]. Several studies showed that CD28 in the CAR construct prolonged proliferation of grafted T cells and increased the secretion of IL-2, IFN-γ, and GM-CSF, independent of exogenous B7/CD28 co-stimulation [34–36].

The CD28-CD3ζ signaling domains of our FOLR1-CAR construct were effective in Jurkat, KHYG-1, and human T cells. Since Jurkat cells are a human immortalized cell line and well-characterized, it would be simpler and more reliable to confirm activation of our FOLR1-CAR construct than using PBMCs (Fig 1). Levels of IL-2 in Jurkat cells and IFN-γ, TNF-α, GM-CSF, and granzyme B in human T cells were increased when FOLR1-CAR cells were co-cultured with FOLR1-positive GC cells (Figs 2C and 7A). In addition, the cytotoxic effects of FOLR1-CAR KHYG-1 and FOLR1-CAR T cells were specific for FOLR1-positive GC cells (Figs 3D and 7B). These results showed that the FOLR1-CAR construct specifically recognizes FOLR1 and the signal is successfully transmitted through the signaling pathway in the cells. In particular, cell signaling is transmitted from CD3ζ to ZAP70, with granzyme B playing an important role in cytotoxicity (Figs 5C and 6A). Xenograft assay also demonstrated that FOLR1-CAR KHYG-1 cells have specific cytotoxicity against FOLR1-positive GC cells (Fig 9). When T cells were co-cultured with KB cells, cell death was observed in mock T cells, though smaller than that in FOLR1-CAR T cells (Fig 5B). When mock T cells were co-cultured with KB cells, western blot analysis revealed that T cell signaling was increased compared to culture without KB cells (Fig 6A). In addition, Z-AAD-CMK reduced target cell apoptosis when mock T cells were co-cultured with KB cells (Fig 5C). These results suggest that TCR reactions occurred in mock T cells and secreted granzyme B to induce target cell apoptosis.

There are various GC cells that are inherently sensitive or insensitive to NK/T cell mediated lysis. FOLR1-positive GC cells (MKN1, MKN7, and GCIY) appeared to be sensitive to NK/T cell mediated lysis compared to the FOLR1-negative GC cells (SNU216, SNU601, and SNU668). Thus, we added SNU484, a FOLR1-positive cell line which is insensitive to NK cells, and SNU638, a FOLR1-negative cell line which is sensitive to NK cells. As shown in Figs 3D, 3E, 7B and 7C, although there was a difference in mock KHYG-1/T cell mediated lysis percentage, cell lysis in FOLR1-CAR KHYG-1/T cells was significantly increased in insensitive SNU484, but not in sensitive SNU638. Therefore, our conclusion is that FOLR1-CAR promote the apoptosis of FOLR1-positive cells.

To evaluate CAR targeting cytotoxic effects, many studies have used NK cell lines, including NK-92, NKG, YT, NK-YS, HANK-1, YTS cells, and NKL cells [37]. The NK cell lines are well-defined and do not require the isolation of NK cells from donors. The NK-92 cell line is the most commonly studied NK cell line and is currently being used in clinical trials [38]. We used the NK cell line KHYG-1 to investigate the cytotoxic effects of CAR because it has several advantages over other NK cell lines. Since the growth rate of KHYG-1 cells is faster than that of other cells, the number of cells required for the experiment can be obtained easily. Doubling times of KHYG-1, NK-92, and YT cells are about 16–18 h, 40–50 h, and 50–60 h, respectively [39, 40]. Several studies recommend that NK-92 cells need to be cultured in special media such as MyeloCult because NK-92 cells grow slowly in RPMI-1640 [41, 42]. Contrary to NK-92 cells, KHYG-1 cells can be grown at a low cost since RPMI-1640 can be used. In addition, the lytic activity of KHYG-1 cells is as effective as that of NK-92 and YT cells against target cells [43, 44]. Given these advantages, KHYG-1 cells are suitable for evaluating the cytotoxicity of CAR.

GC is one of the most difficult malignancies to treat, and targeted therapies for GC are constantly being developed. Several studies have shown that FOLR1 is a good target for various cancers [24]. We showed that FOLR1-CAR T cells exhibited specific recognition and effective anticancer activity against FOLR1-positive GC cells. Therefore, our results suggest that our FOLR1-CAR T cells are an excellent option for treating in patients who have FOLR1-positive GC cells.

## Supporting information

S1 Fig Specific activity of FOLR1-CAR KHYG-1 cells. KB cells staining with 10 μM of Calcein-AM for 30 minutes. KB cells were co-cultured with mock KHYG-1 cells (A) or FOLR1-CAR KHYG-1 cells (B) at E/T ratio of 10:1. The pictures were taken every 2 hours.
(TIF)

S2 Fig Specific activity of FOLR1-CAR T cells. KB cells staining with 10 μM of Calcein-AM for 30 minutes. KB cells were co-cultured with mock T cells (A) or FOLR1-CAR T cells (B) at E/T ratio of 10:1. The pictures were taken every 2 hours.
(TIF)

S1 Table Antibody information. (DOCX)

## Abbreviations

CAR: chimeric antigen receptor
CEA: carcinoembryonic antigen
EGFR: epidermal growth factor receptor
ELISA: enzyme-linked immunosorbent assay
EpCAM: epithelial cell adhesion molecule
FACS: fluorescence-activated cell sorting
FOLR1: folate receptor 1
GC: gastric cancer
GM-CSF: granulocyte-macrophage colony-stimulating factor
HER2: human epidermal growth factor receptor 2
IFN-γ: interferon- γ
IL-2: interleukin-2
KB cell line: a subline of the ubiquitous keratin forming tumor cell line HeLa
MHC: major histocompatibility complex
MSLN: mesothelin
NK: natural killer
PBMC: peripheral blood mononuclear cells
scFv: single-chain variable fragment
TCR: T cell receptor
TNF-α: tumor necrosis factor- α
ZAP70: zeta-chain-associated protein kinase 70

## References

[1] Gao J, Bernatchez C, Sharma P, Radvanyi LG, Hwu P. Advances in the development of cancer immunotherapies. Trends Immunol. 2013;34(2):90–8. doi: 10.1016/j.it.2012.08.004 . 23031830 10.1016/j.it.2012.08.004 PMC3565019

[2] Maus MV, Fraietta JA, Levine BL, Kalos M, Zhao Y, June CH. Adoptive immunotherapy for cancer or viruses. Annu Rev Immunol. 2014;32:189–225. doi: 10.1146/annurev-immunol-032713-120136 . 24423116 10.1146/annurev-immunol-032713-120136 PMC4533835

[3] Maher J. Immunotherapy of malignant disease using chimeric antigen receptor engrafted T cells. ISRN Oncol. 2012;2012:278093 doi: 10.5402/2012/278093 . 23304553 10.5402/2012/278093 PMC3523553

[4] Finney HM, Lawson AD, Bebbington CR, Weir AN. Chimeric receptors providing both primary and costimulatory signaling in T cells from a single gene product. J Immunol. 1998;161(6):2791–7. . 9743337

[5] Cartellieri M, Bachmann M, Feldmann A, Bippes C, Stamova S, Wehner R, et al Chimeric antigen receptor-engineered T cells for immunotherapy of cancer. J Biomed Biotechnol. 2010;2010:956304 doi: 10.1155/2010/956304 . 20467460 10.1155/2010/956304 PMC2864912

[6] Holzinger A, Barden M, Abken H. The growing world of CAR T cell trials: a systematic review. Cancer Immunol Immunother. 2016;65(12):1433–50. doi: 10.1007/s00262-016-1895-5 . 27613725 10.1007/s00262-016-1895-5 PMC11029082

[7] Kakarla S, Gottschalk S. CAR T cells for solid tumors: armed and ready to go? Cancer J. 2014;20(2):151–5. doi: 10.1097/PPO.0000000000000032 . 24667962 10.1097/PPO.0000000000000032 PMC4050065

[8] Yu S, Li A, Liu Q, Li T, Yuan X, Han X, et al Chimeric antigen receptor T cells: a novel therapy for solid tumors. J Hematol Oncol. 2017;10(1):78 doi: 10.1186/s13045-017-0444-9 . 28356156 10.1186/s13045-017-0444-9 PMC5372296

[9] Vergote IB, Marth C, Coleman RL. Role of the folate receptor in ovarian cancer treatment: evidence, mechanism, and clinical implications. Cancer Metastasis Rev. 2015;34(1):41–52. doi: 10.1007/s10555-014-9539-8 . 25564455 10.1007/s10555-014-9539-8

[10] Elnakat H, Ratnam M. Role of folate receptor genes in reproduction and related cancers. Front Biosci. 2006;11:506–19. . 16146749 10.2741/1815

[11] Rahman R, Asombang AW, Ibdah JA. Characteristics of gastric cancer in Asia. World J Gastroenterol. 2014;20(16):4483–90. doi: 10.3748/wjg.v20.i16.4483 . 24782601 10.3748/wjg.v20.i16.4483 PMC4000485

[12] Low PS, Kularatne SA. Folate-targeted therapeutic and imaging agents for cancer. Curr Opin Chem Biol. 2009;13(3):256–62. doi: 10.1016/j.cbpa.2009.03.022 . 19419901 10.1016/j.cbpa.2009.03.022

[13] Kershaw MH, Westwood JA, Parker LL, Wang G, Eshhar Z, Mavroukakis SA, et al A phase I study on adoptive immunotherapy using gene-modified T cells for ovarian cancer. Clin Cancer Res. 2006;12(20 Pt 1):6106–15. doi: 10.1158/1078-0432.CCR-06-1183 . 17062687 10.1158/1078-0432.CCR-06-1183 PMC2154351

[14] Barry M, Bleackley RC. Cytotoxic T lymphocytes: all roads lead to death. Nat Rev Immunol. 2002;2(6):401–9. doi: 10.1038/nri819 . 12093006 10.1038/nri819

[15] Wang H, Kadlecek TA, Au-Yeung BB, Goodfellow HE, Hsu LY, Freedman TS, et al ZAP-70: an essential kinase in T-cell signaling. Cold Spring Harb Perspect Biol. 2010;2(5):a002279 doi: 10.1101/cshperspect.a002279 . 20452964 10.1101/cshperspect.a002279 PMC2857167

[16] Rousalova I, Krepela E. Granzyme B-induced apoptosis in cancer cells and its regulation (review). Int J Oncol. 2010;37(6):1361–78. Epub 2010/11/03. . 21042704 10.3892/ijo_00000788

[17] Zhang Q, Zhang Z, Peng M, Fu S, Xue Z, Zhang R. CAR-T cell therapy in gastrointestinal tumors and hepatic carcinoma: From bench to bedside. Oncoimmunology. 2016;5(12):e1251539 doi: 10.1080/2162402X.2016.1251539 . 28123893 10.1080/2162402X.2016.1251539 PMC5214859

[18] Abrahao-Machado LF, Scapulatempo-Neto C. HER2 testing in gastric cancer: An update. World J Gastroenterol. 2016;22(19):4619–25. doi: 10.3748/wjg.v22.i19.4619 . 27217694 10.3748/wjg.v22.i19.4619 PMC4870069

[19] Kobold S, Steffen J, Chaloupka M, Grassmann S, Henkel J, Castoldi R, et al Selective bispecific T cell recruiting antibody and antitumor activity of adoptive T cell transfer. J Natl Cancer Inst. 2015;107(1):364 doi: 10.1093/jnci/dju364 . 25424197 10.1093/jnci/dju364

[20] Maher J, Wilkie S. CAR mechanics: driving T cells into the MUC of cancer. Cancer Res. 2009;69(11):4559–62. doi: 10.1158/0008-5472.CAN-09-0564 . 19487277 10.1158/0008-5472.CAN-09-0564

[21] Warneke VS, Behrens HM, Haag J, Kruger S, Simon E, Mathiak M, et al Members of the EpCAM signalling pathway are expressed in gastric cancer tissue and are correlated with patient prognosis. Br J Cancer. 2013;109(8):2217–27. doi: 10.1038/bjc.2013.536 . 24008668 10.1038/bjc.2013.536 PMC3798952

[22] Boshnjaku V, Shim KW, Tsurubuchi T, Ichi S, Szany EV, Xi G, et al Nuclear localization of folate receptor alpha: a new role as a transcription factor. Sci Rep. 2012;2:980 doi: 10.1038/srep00980 . 23243496 10.1038/srep00980 PMC3522071

[23] Siu MK, Kong DS, Chan HY, Wong ES, Ip PP, Jiang L, et al Paradoxical impact of two folate receptors, FRalpha and RFC, in ovarian cancer: effect on cell proliferation, invasion and clinical outcome. PLoS One. 2012;7(11):e47201 doi: 10.1371/journal.pone.0047201 . 23144806 10.1371/journal.pone.0047201 PMC3492371

[24] Cheung A, Bax HJ, Josephs DH, Ilieva KM, Pellizzari G, Opzoomer J, et al Targeting folate receptor alpha for cancer treatment. Oncotarget. 2016;7(32):52553–74. doi: 10.18632/oncotarget.9651 . 27248175 10.18632/oncotarget.9651 PMC5239573

[25] Dosio F, Milla P, Cattel L. EC-145, a folate-targeted Vinca alkaloid conjugate for the potential treatment of folate receptor-expressing cancers. Curr Opin Investig Drugs. 2010;11(12):1424–33. . 21154124

[26] Kamen BA, Smith AK. Farletuzumab, an anti-folate receptor alpha antibody, does not block binding of folate or anti-folates to receptor nor does it alter the potency of anti-folates in vitro. Cancer Chemother Pharmacol. 2012;70(1):113–20. doi: 10.1007/s00280-012-1890-2 . 22644798 10.1007/s00280-012-1890-2

[27] Lin J, Spidel JL, Maddage CJ, Rybinski KA, Kennedy RP, Krauthauser CL, et al The antitumor activity of the human FOLR1-specific monoclonal antibody, farletuzumab, in an ovarian cancer mouse model is mediated by antibody-dependent cellular cytotoxicity. Cancer Biol Ther. 2013;14(11):1032–8. doi: 10.4161/cbt.26106 . 24025360 10.4161/cbt.26106 PMC3925658

[28] Wen Y, Graybill WS, Previs RA, Hu W, Ivan C, Mangala LS, et al Immunotherapy targeting folate receptor induces cell death associated with autophagy in ovarian cancer. Clin Cancer Res. 2015;21(2):448–59. doi: 10.1158/1078-0432.CCR-14-1578 . 25416196 10.1158/1078-0432.CCR-14-1578 PMC4297546

[29] Eshhar Z, Bach N, Fitzer-Attas CJ, Gross G, Lustgarten J, Waks T, et al The T-body approach: potential for cancer immunotherapy. Springer Semin Immunopathol. 1996;18(2):199–209. . 8908700 10.1007/BF00820666

[30] Weijtens ME, Willemsen RA, Valerio D, Stam K, Bolhuis RL. Single chain Ig/gamma gene-redirected human T lymphocytes produce cytokines, specifically lyse tumor cells, and recycle lytic capacity. J Immunol. 1996;157(2):836–43. . 8752936

[31] Haynes NM, Snook MB, Trapani JA, Cerruti L, Jane SM, Smyth MJ, et al Redirecting mouse CTL against colon carcinoma: superior signaling efficacy of single-chain variable domain chimeras containing TCR-zeta vs Fc epsilon RI-gamma. J Immunol. 2001;166(1):182–7. . 11123291 10.4049/jimmunol.166.1.182

[32] Heuser C, Hombach A, Losch C, Manista K, Abken H. T-cell activation by recombinant immunoreceptors: impact of the intracellular signalling domain on the stability of receptor expression and antigen-specific activation of grafted T cells. Gene Ther. 2003;10(17):1408–19. doi: 10.1038/sj.gt.3302023 . 12900755 10.1038/sj.gt.3302023

[33] Brentjens RJ, Santos E, Nikhamin Y, Yeh R, Matsushita M, La Perle K, et al Genetically targeted T cells eradicate systemic acute lymphoblastic leukemia xenografts. Clin Cancer Res. 2007;13(18 Pt 1):5426–35. doi: 10.1158/1078-0432.CCR-07-0674 . 17855649 10.1158/1078-0432.CCR-07-0674

[34] Beecham EJ, Ma Q, Ripley R, Junghans RP. Coupling CD28 co-stimulation to immunoglobulin T-cell receptor molecules: the dynamics of T-cell proliferation and death. J Immunother. 2000;23(6):631–42. . 11186151 10.1097/00002371-200011000-00004

[35] Hombach A, Sent D, Schneider C, Heuser C, Koch D, Pohl C, et al T-cell activation by recombinant receptors: CD28 costimulation is required for interleukin 2 secretion and receptor-mediated T-cell proliferation but does not affect receptor-mediated target cell lysis. Cancer Res. 2001;61(5):1976–82. . 11280755

[36] Haynes NM, Trapani JA, Teng MW, Jackson JT, Cerruti L, Jane SM, et al Rejection of syngeneic colon carcinoma by CTLs expressing single-chain antibody receptors codelivering CD28 costimulation. J Immunol. 2002;169(10):5780–6. . 12421958 10.4049/jimmunol.169.10.5780

[37] Hermanson DL, Kaufman DS. Utilizing chimeric antigen receptors to direct natural killer cell activity. Front Immunol. 2015;6:195 doi: 10.3389/fimmu.2015.00195 . 25972867 10.3389/fimmu.2015.00195 PMC4412125

[38] Tonn T, Schwabe D, Klingemann HG, Becker S, Esser R, Koehl U, et al Treatment of patients with advanced cancer with the natural killer cell line NK-92. Cytotherapy. 2013;15(12):1563–70. doi: 10.1016/j.jcyt.2013.06.017 . 24094496 10.1016/j.jcyt.2013.06.017

[39] Taguchi Y, Kondo T, Watanabe M, Miyaji M, Umehara H, Kozutsumi Y, et al Interleukin-2-induced survival of natural killer (NK) cells involving phosphatidylinositol-3 kinase-dependent reduction of ceramide through acid sphingomyelinase, sphingomyelin synthase, and glucosylceramide synthase. Blood. 2004;104(10):3285–93. doi: 10.1182/blood-2004-03-0900 . 15271800 10.1182/blood-2004-03-0900

[40] Gross C, Schmidt-Wolf IG, Nagaraj S, Gastpar R, Ellwart J, Kunz-Schughart LA, et al Heat shock protein 70-reactivity is associated with increased cell surface density of CD94/CD56 on primary natural killer cells. Cell Stress Chaperones. 2003;8(4):348–60. . 15115287 10.1379/1466-1268(2003)008<0348:hspria>2.0.co;2 PMC514906

[41] Boissel L, Betancur-Boissel M, Lu W, Krause DS, Van Etten RA, Wels WS, et al Retargeting NK-92 cells by means of CD19- and CD20-specific chimeric antigen receptors compares favorably with antibody-dependent cellular cytotoxicity. Oncoimmunology. 2013;2(10):e26527 doi: 10.4161/onci.26527 . 24404423 10.4161/onci.26527 PMC3881109

[42] Schonfeld K, Sahm C, Zhang C, Naundorf S, Brendel C, Odendahl M, et al Selective inhibition of tumor growth by clonal NK cells expressing an ErbB2/HER2-specific chimeric antigen receptor. Mol Ther. 2015;23(2):330–8. doi: 10.1038/mt.2014.219 . 25373520 10.1038/mt.2014.219 PMC4445620

[43] Suck G, Branch DR, Smyth MJ, Miller RG, Vergidis J, Fahim S, et al KHYG-1, a model for the study of enhanced natural killer cell cytotoxicity. Exp Hematol. 2005;33(10):1160–71. doi: 10.1016/j.exphem.2005.06.024 . 16219538 10.1016/j.exphem.2005.06.024

[44] Klingemann H, Boissel L, Toneguzzo F. Natural Killer Cells for Immunotherapy—Advantages of the NK-92 Cell Line over Blood NK Cells. Front Immunol. 2016;7:91 doi: 10.3389/fimmu.2016.00091 . 27014270 10.3389/fimmu.2016.00091 PMC4789404

