# Supplementary figures and images for: Folate receptor 1 (FOLR1) targeted chimeric antigen receptor (CAR) T cells for the treatment of gastric cancer
Source: PLoS One. 2018 Jun 6;13(6):e0198347. doi: 10.1371/journal.pone.0198347 (PMC5991383; doi:10.1371/journal.pone.0198347)

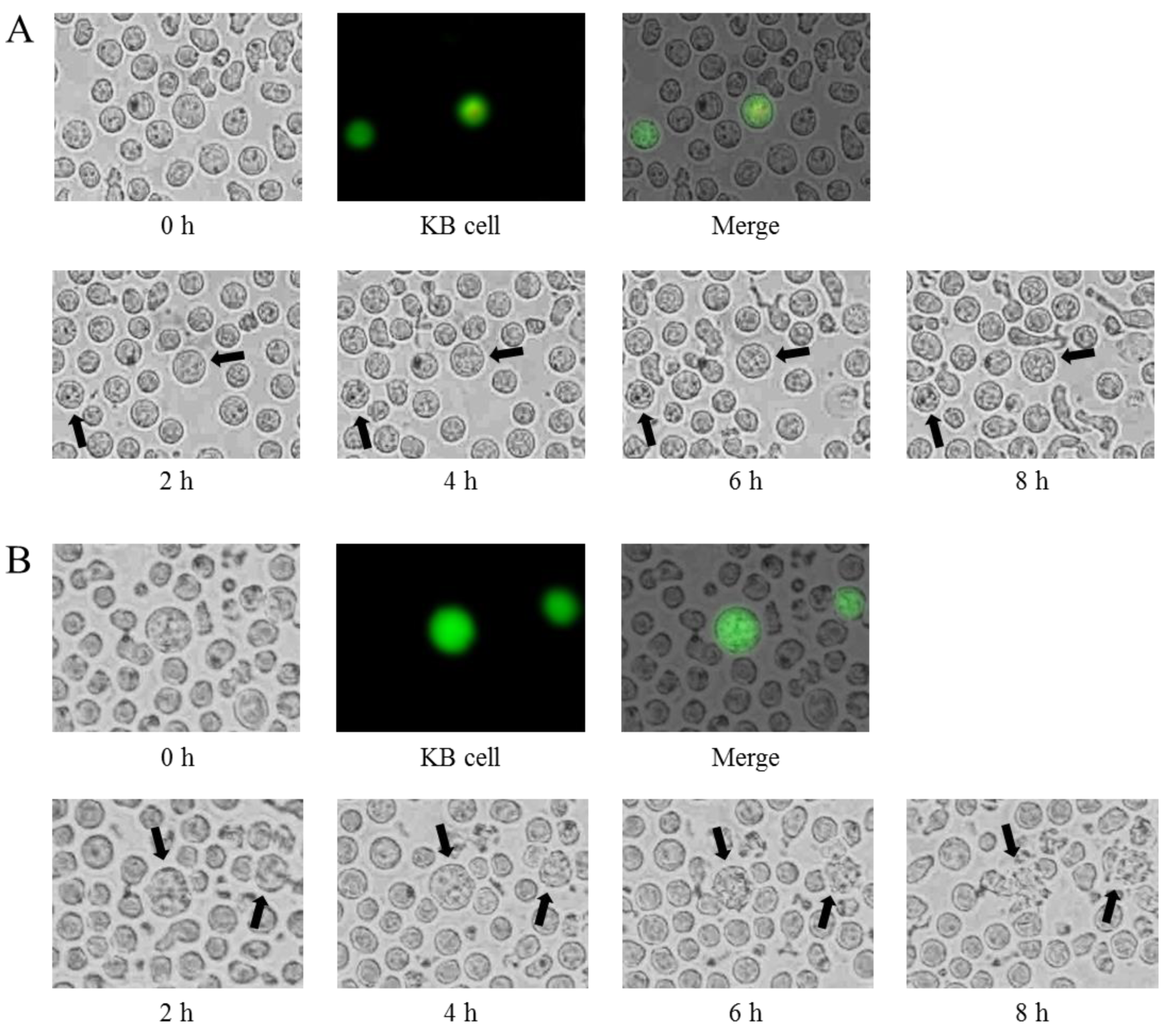

Supplement: S1 Fig — KB cells staining with 10 μM of Calcein-AM for 30 minutes. KB cells were co-cultured with mock KHYG-1 cells (A) or FOLR1-CAR KHYG-1 cells (B) at E/T ratio of 10:1. The pictures were taken every 2 hours. (TIF) [file pone.0198347.s001.tif]

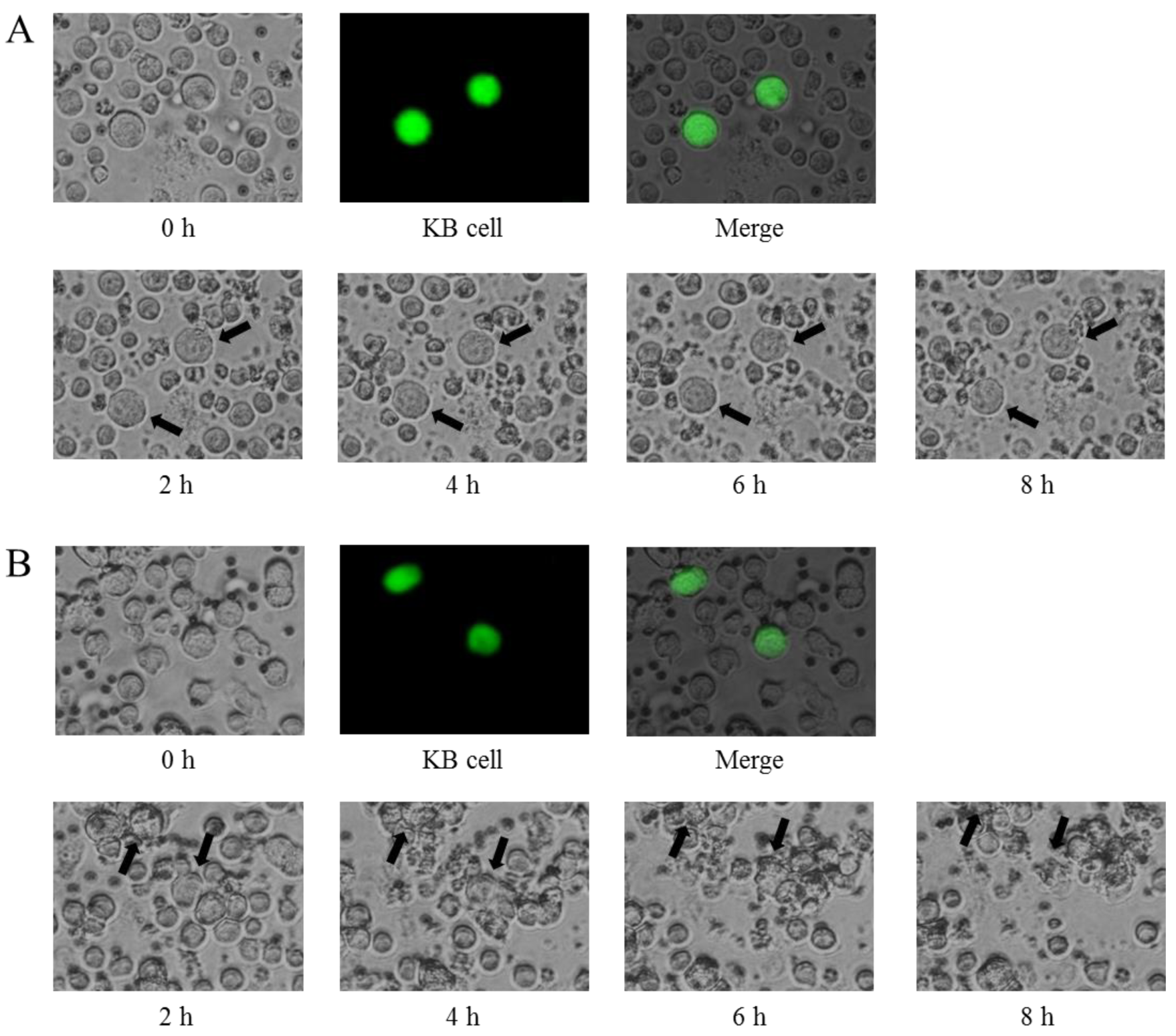

Supplement: S2 Fig — KB cells staining with 10 μM of Calcein-AM for 30 minutes. KB cells were co-cultured with mock T cells (A) or FOLR1-CAR T cells (B) at E/T ratio of 10:1. The pictures were taken every 2 hours. (TIF) [file pone.0198347.s002.tif]
