# Supplementary material for: Folate receptor 1 (FOLR1) targeted chimeric antigen receptor (CAR) T cells for the treatment of gastric cancer
Source: PLoS One. 2018 Jun 6;13(6):e0198347. doi: 10.1371/journal.pone.0198347 (PMC5991383; doi:10.1371/journal.pone.0198347)
Supplement: S1 Table — (DOCX) [file pone.0198347.s003.docx]

**S1 Table. Antibody information**

| Antibody | Company | Cat. no |
| --- | --- | --- |
| GAPDH | Cell Signaling Technology | #5174S |
| α-Tubulin | Cell Signaling Technology | #3873S |
| Myc-tag | Origene Technologies | #TA150121 |
| CD3ζ | BD Biosciences | #551034 |
| ZAP70 | Cell Signaling Technology | #2709S |
| p-ZAP70 | Cell Signaling Technology | #2717T |
| AKT | Santa Cruz | #sc-1618 |
| p-AKT | Santa Cruz | #sc-7985-R |
| JNK | Cell Signaling Technology | #9252S |
| p-JNK | Cell Signaling Technology | #9255S |
| p38 | Cell Signaling Technology | #9212S |
| p-p38 | Cell Signaling Technology | #9211S |
| ERK | Cell Signaling Technology | #4695S |
| p-ERK | Cell Signaling Technology | #4370S |
| tBid | Cell Signaling Technology | #2002S |
| PARP | Cell Signaling Technology | #9542S |
| Caspase 8 | BioLegend | #622001 |
| Caspase 9 | BioLegend | #621901 |
| Caspase 3 | Cell Signaling Technology | #9664S |
| Normal IgG (mouse) | Santa Cruz | #sc-2025 |
| Normal IgG (rabbit) | Cell Signaling Technology | #2729S |
| FOLR1 | Abcam | #ab3361 |
| CD3 | Abcam | #ab34182 |
| CD4 | Abcam | #ab133616 |
| CD8 | Cell Signaling Technology | #ab4055 |
| Anti-mouse Alexa 488 | Thermo Fisher Scientific | #A-110011 |
| Anti-rabbit Alexa 647 | Abcam | #ab150079 |
